# Validation of the portuguese version of the mentalization questionnaire in a sample of college students: Preliminary results

**DOI:** 10.1192/j.eurpsy.2021.1184

**Published:** 2021-08-13

**Authors:** P. Rebelo, B. Gooskens, D. Pereira, C. Cabaços, A.T. Pereira, S. Caldeira, N. Madeira, S. Bos

**Affiliations:** 1 Faculty Of Medicine, University of Coimbra, Coimbra, Portugal; 2 Institute Of Psychological Medicine, Faculty of Medicine – University of Coimbra, Coimbra, Portugal; 3 Psychiatry, Coimbra Hospital and University Centre, Coimbra, Portugal

**Keywords:** Mentalization Questionnaire, Portuguese Version, psychometric properties, Reliability

## Abstract

**Introduction:**

Mentalization (MZ) is the capacity by which people make sense of their own’s and others’ mental states; when compromised, it is associated with several mental disorders (Bateman & Fonagy, 2010). A valid instrument to assess MZ is missing and required for the Portuguese population.

**Objectives:**

To develop and validate a Portuguese version (Questionário de Mentalização – QMZ) of the Mentalization Questionnaire (MZQ) (Hausberg et al., 2012).

**Methods:**

A sample of 184 Portuguese medical students (mean age = 21.6 ± 2.47 years, 59.8% female) was used to explore the psychometric properties of the scale, using reliability and factor analysis (varimax rotation method).

**Results:**

The QMZ exhibited a Cronbach’s alpha score of .80. All items contributed to its reliability. Based on the scree plot of Cattell and interpretability of items, a 2-factor and a 5-factor structures were further explored. The former explained 38.8% of the total variance (VE) and included a regulation of affect (VE= 27.3%, α=.79) and a self-reflection and emotional awareness (VE= 11.5%, α=.62) factors. The latter explained 60.6% of the total variance and incorporated the following dimensions: self-control (VE= 27.3%, α=.74), daily relationships (VE= 11.5%, α=.67), self-comprehension (VE= 8.1%, α=.54), close relationships (VE= 7.0%, α=.41) and self-monitoring (VE= 6.7%, α=.52). The 5-factor structure was closer to the dimensional concept of mentalization.

**Conclusions:**

The QMZ has proved to be a promising instrument, with adequate psychometric properties, confirmed by its acceptable construct, criterion and factorial validity and reliability to assess mentalization in Portuguese language.

